# CD301b^+^ monocyte-derived dendritic cells mediate resistance to radiotherapy

**DOI:** 10.1084/jem.20231717

**Published:** 2025-03-27

**Authors:** Sirimuvva Tadepalli, Derek R. Clements, Hayley M. Raquer-McKay, Anja Lüdtke, Sanjana Saravanan, David Seong, Lorraine Vitek, Christopher M. Richards, Jan E. Carette, Matthias Mack, Andres Gottfried-Blackmore, Edward E. Graves, Juliana Idoyaga

**Affiliations:** 1 Department of Microbiology and Immunology, Stanford University School of Medicine, Stanford, CA, USA; 2 Immunology Program, Stanford University School of Medicine, Stanford, CA, USA; 3 Department of Radiation Oncology, Molecular Imaging Program at Stanford, Stanford University School of Medicine, Stanford, CA, USA; 4 Stanford Medical Scientist Training Program, Stanford University School of Medicine, Stanford, CA, USA; 5 Department of Nephrology, University Hospital Regensburg, Regensburg, Germany; 6 Department of Pharmacology, University of California San Diego School of Medicine, San Diego, CA, USA; 7 Department of Medicine, Division of Gastroenterology, University of California San Diego School of Medicine, San Diego, CA, USA; 8 Gastroenterology Section, Veterans Affairs San Diego Healthcare System, San Diego, CA, USA; 9 Department of Molecular Biology, University of California San Diego School of Biological Sciences, San Diego, CA, USA

## Abstract

Monocytes infiltrating tumors acquire various states that distinctly impact cancer treatment. Here, we show that resistance of tumors to radiotherapy (RT) is controlled by the accumulation of monocyte-derived dendritic cells (moDCs). These moDCs are characterized by the expression of CD301b and have a superior capacity to generate regulatory T cells (Tregs). Accordingly, moDC depletion limits Treg generation and improves the therapeutic outcome of RT. Mechanistically, we demonstrate that granulocyte–macrophage colony-stimulating factor (GM-CSF) derived from radioresistant tumor cells following RT is necessary for the accumulation of moDCs. Our results unravel the immunosuppressive function of moDCs and identify GM-CSF as an immunotherapeutic target during RT.

## Teaser

Radiotherapy not only kills tumors but also shapes immunity. This study reveals that in radioresistant tumors, clinically relevant radiotherapy induces GM-CSF secretion, driving immunosuppressive monocyte-derived dendritic cells and regulatory T cells. Targeting monocyte differentiation and GM-CSF may enhance radiotherapy efficacy.

## Graphical Abstract

**Figure:**
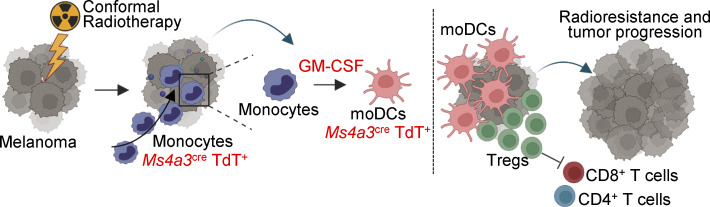

## Introduction

Radiotherapy (RT) plays a central role in cancer treatment, as it is used in over 50% of all patients (Citrin, 2017). Besides its well-documented cytotoxic effect in cancer cells, RT modulates the immune system, resulting in either immunostimulatory or immunosuppressive responses that ultimately determine treatment efficacy (Golden and Formenti, 2014; Deng et al., 2014b; Liang et al., 2017; Ostrand-Rosenberg et al., 2019; Lynch et al., 2024). Unraveling RT-induced immune mechanisms could reveal emerging avenues for combinatory treatments.

One well-characterized immune consequence of RT is the recruitment of monocytes to the tumor microenvironment (TME) (Xu et al., 2013; Liang et al., 2017). In the TME, monocytes can differentiate into immunosuppressive cells, and consequently, monocytes and their progeny are commonly categorized as myeloid-derived suppressor cells (MDSCs) (Gabrilovich and Nagaraj, 2009). Although this classification initially highlighted the suppressive function of myeloid cells during cancer treatment, it is now well accepted that MDSCs are highly heterogeneous (Hegde et al., 2021). Dissecting this heterogeneity and linking cellular origins to distinct functional states could provide precise therapeutic targets. This can be achieved through high-dimensional single-cell approaches and fate-tracing models. By leveraging these technologies, we and others have tracked monocyte fates following cancer treatments, identifying discrete populations with either immunostimulatory (Kwart et al., 2022; Tadepalli et al., 2023) or immunosuppressive (Dunsmore et al., 2024; Hegde et al., 2024, *Preprint*) functions. Importantly, defining these populations allows to unravel signaling pathways that govern their differentiation, offering potential therapeutic insights. One such pathway involves granulocyte–macrophage colony-stimulating factor (GM-CSF), a growth factor that drives myeloid cell generation, including monocytes (Ushach and Zlotnik, 2016; Ribechini et al., 2017). Interestingly, GM-CSF is reported to either suppress or promote tumor progression (Hong, 2016; Kumar et al., 2022), necessitating a deeper understanding of the conditions in which this growth factor mediates beneficial versus detrimental functions during cancer therapy.

Most of our knowledge of monocyte and monocyte-derived cell function after RT comes from mouse preclinical approaches. Until recently, these approaches primarily used shielding techniques to protect small animals from systemic irradiation (hereafter called shielded RT). However, this approach differs significantly from clinically applied conformal RT, which utilizes volumetric computed tomography (CT) scans to map tumors and precisely deliver high radiation doses while sparing healthy tissues. Recent technological advancements now allow the delivery of conformal RT to small-animal tumors, better modeling patient treatment (Bazalova et al., 2014). Importantly, we have recently reported that immune responses to conformal RT differ from those triggered by shielded RT (Tadepalli et al., 2023). In a radiosensitive tumor model, conformal RT, but not shielded RT, promotes monocyte activation via type I interferon (IFN-I) signaling (Tadepalli et al., 2023). This IFN-I–driven activated monocyte (mono^ACT^) state enhances CD8^+^ T cell effector function, leading to tumor elimination. However, the fate of monocytes in tumors resistant to RT remains unexplored. Notably, previous studies have linked resistance to shielded RT with the accumulation of FOXP3^+^ regulatory T cells (Tregs) (Kachikwu et al., 2011; Schuler et al., 2013; Wirsdörfer et al., 2014; Twyman-Saint Victor et al., 2015), which suppress dendritic cell (DC) and T cell function (Jang et al., 2017; Zagorulya et al., 2023). Whether this immunosuppressive mechanism also applies to conformal RT is unknown. Identifying resistance mechanisms in the context of conformal RT would facilitate the rational design of combinatory therapies with clinical relevance.

Here, using high-dimensional single-cell approaches and fate-tracing experiments, we investigated the mononuclear phagocyte population mediating resistance to clinically relevant conformal RT. We found that conformal RT induces the infiltration of monocytes into the TME, followed by their proliferation and differentiation into CD301b^+^ monocyte–derived DCs (moDCs). These moDCs, identifiable through a monocyte-specific fate-tracing mouse model, are otherwise phenotypically and transcriptionally similar to DC type 2 (DC2s), suggesting that environmental cues, rather than cell origin, determine their features. We show that CD301b^+^ moDCs preferentially induce Tregs, and their depletion enhances RT responses. Mechanistically, we found that tumor-derived GM-CSF drives CD301b^+^ moDC accumulation after RT. Our study highlights the immunosuppressive role of moDCs in Treg induction during clinically relevant conformal RT and identifies tumor-derived GM-CSF as a promising therapeutic target.

## Results

### Resistance of melanoma to conformal RT is mediated by Tregs

**Figure 1.**
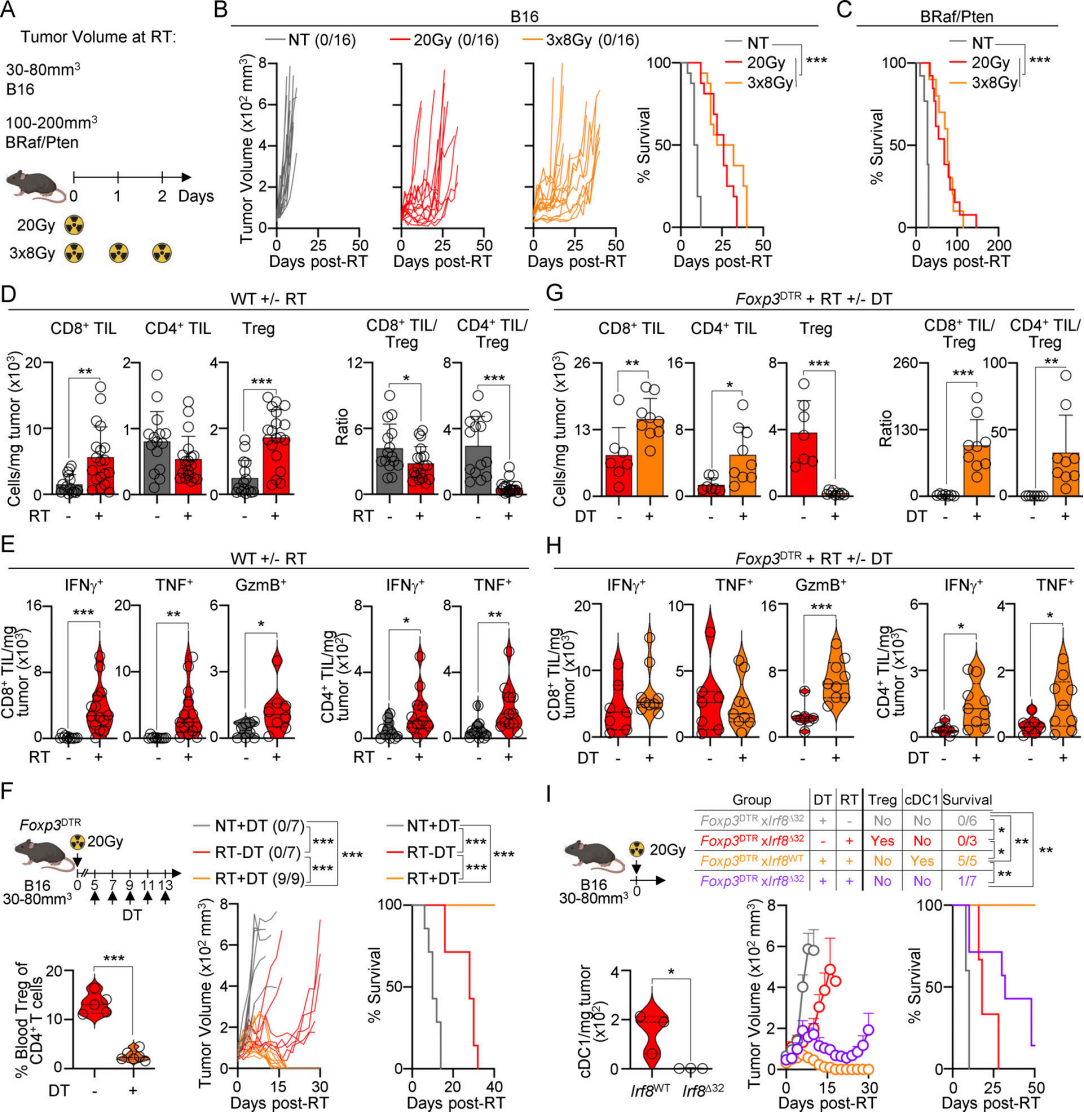
**Treg accumulation mediates tumor resistance to RT. (A)** B16 tumors were implanted subcutaneously in B6 mice, and BRaf/Pten tumors were induced by 4-hydroxytamoxifen. Tumors were treated with 20 Gy or three 8 Gy (3 × 8 Gy) doses of RT at the indicated tumor sizes. **(B)** Left: individual B16 tumor growth with a ratio of surviving mice in parentheses. Right: survival curves (*n* = 16/group, 3–5 experiments [exp.]). **(C)** Survival curves for BRaf/Pten tumors (*n* = 10–13/group, 2–4 exp.). **(D and E)** B16 tumors were treated or not with 20 Gy RT. **(D)** TIL numbers 7 days after RT, by flow cytometry (mean + SD; *n* = 14–19/group, 3–5 exp.). **(E)** TILs producing IFNγ, TNF, and GzmB 7 days after RT, after ex vivo restimulation, by flow cytometry (violin plots show data distribution; *n* = 8–11/3–4 exp.). **(F–H)** B16-bearing *Foxp3*^DTR^ mice were treated or not with 20 Gy RT and DT. **(F)** Left: frequency of blood Treg within CD4^+^ T cells (violin plots show data distribution; *n* = 5–6/group, 2 exp.). Right: individual tumor growth (with a ratio of surviving mice in parentheses) and survival curves (*n* = 7–9/group, 3 exp.). **(G)** TIL numbers 7 days after RT, by flow cytometry (mean + SD; *n* = 7–9/group, 4 exp.). **(H)** TILs producing IFNγ, TNF, and GzmB 7 days after RT, after ex vivo restimulation, by flow cytometry (violin plots show data distribution; *n* = 7–9/group, 4 exp.). **(I)** Left: tumor-infiltrating cDC1 numbers in *Irf8*^WT^ and *Irf8*^Δ32^ mice 7 days after RT, by flow cytometry (violin plots show data distribution; *n* = 3/group, 2 exp.). Right: average tumor growth (mean + SEM) and survival of mice treated or not with RT and DT (*n* = 3–7/group, 3 exp.). Statistics: two-way ANOVA plus Tukey’s post hoc test for mean tumor growth (B, F, and I); Mantel–Cox’s test for survival curves (B, C, F, and I); two-tailed unpaired *t* test for the rest. *P ≤ 0.05, **P ≤ 0.01, ***P ≤ 0.001.

Melanoma is considered a radioresistant tumor (Espenel et al., 2017); however, the immune mechanisms underlying this resistance have only been analyzed using shielded RT and not clinically relevant conformal RT (Liang et al., 2017). To investigate immune responses after conformal RT, we established two melanoma tumors, i.e., a subcutaneously engrafted B16F1 (B16) and a genetically induced *Braf*^*CA*^xPten^f/f^xTyr^CreER^ (BRaf/Pten) tumors. Conformal RT was delivered using an X-RAD SmART system, which limits damage to healthy tissues through CT-guided imaging (Koontz et al., 2017). Conformal RT was administered as either a single ablative 20 Gy dose or three fractionated 8 Gy doses over three consecutive days (3 × 8 Gy) (Fig. 1 A). RT delayed tumor growth and significantly prolonged survival compared with nontreated mice in both tumor models (Fig. 1, B and C). However, regardless of the fractionation regimen, tumors eventually progressed, and mice succumbed to the disease, showing resistance to treatment. Since no difference was observed in primary tumor growth between the fractionation regimens (Fig. 1, B and C), we continued our study using a single ablative 20 Gy dose.

**Figure S1.**
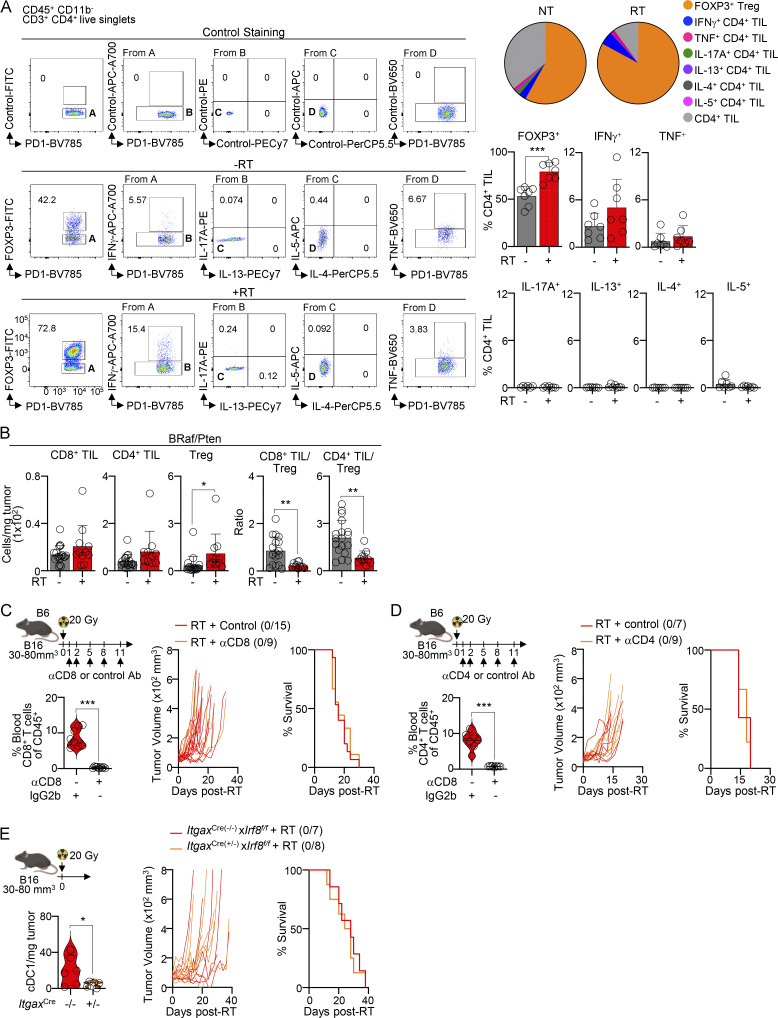
**T cell analysis after RT. (A)** B16-bearing B6 mice were treated or not with 20 Gy RT, and tumors were analyzed 7 days later, after ex vivo restimulation, by flow cytometry. Left: representative flow cytometry plots. Right: frequency of CD4^+^ TILs (mean + SD; *n* = 7/group, 3–4 experiments [exp.]). **(B)** BRaf/Pten tumors were treated or not with 20 Gy RT. TIL numbers and TIL-to-Treg ratio 7 days after RT, by flow cytometry (mean + SD; *n* = 11–18/group, 3–5 exp.). **(C)** B16 tumors treated with 20 Gy RT followed by CD8^+^ T cell depletion. Left: frequency of blood CD8^+^ T cells (violin plots show data distribution; *n* = 8–9/group, 2 exp.). Right: individual tumor growth (with a ratio of surviving mice in parentheses) and survival curves (*n* = 9–15/group, 2–3 exp.). **(D)** Same as C but depleting CD4^+^ T cells. Left: frequency of blood CD4^+^ T cells (violin plots show data distribution; *n* = 7–9/group, 2 exp.). Right: individual tumor growth (with a ratio of surviving mice in parentheses) and survival curves (*n* = 7–9/group, 2 exp.). **(E)** B16-bearing *Itgax-*Cre × *Irf8*^*f/f*^ mice were treated with 20 Gy RT. Left: tumor-infiltrating cDC1 numbers at endpoint (violin plots show data distribution; *n* = 6/group, 2 exp.). Right: individual tumor growth (with a ratio of surviving mice in parentheses) and survival curves (*n* = 7–8/group, 2 exp.). Statistics: two-way ANOVA plus Tukey’s post hoc test for mean tumor growth (C–E); Mantel–Cox’s test for survival curves (C–E); two-tailed *t* test for the rest. *P ≤ 0.05, **P ≤ 0.01, ***P ≤ 0.001.

To evaluate the mechanisms of RT resistance, we first analyzed tumor-infiltrating lymphocytes (TILs). RT induced an increase in the number and function of CD8^+^ TILs (IFNγ^+^ TNF^+^ and Granzyme^+^; Fig. 1, D and E), as well as IFNγ^+^ and TNF^+^ CD4^+^ TILs, but not IL-17^+^, IL-13^+^, IL-4^+^, or IL-5^+^ CD4^+^ T cells (Fig. 1, D and E; and Fig. S1 A). Unlike treatment of radiosensitive MC38 tumors (Tadepalli et al., 2023), RT led to an increase in the numbers of FOXP3^+^ Tregs, resulting in a decreased TIL/Treg ratio in both B16 and BRaf/Pten melanoma tumors (Fig. 1 D and Fig. S1 B). Depleting either CD8^+^ or CD4^+^ TILs did not affect RT efficacy (Fig. S1, C and D). Conversely, depletion of Tregs by diphtheria toxin (DT) inoculation in *Foxp3*^DTR^ mice resulted in complete tumor elimination after RT (Fig. 1 F), which correlated with enhanced TIL numbers and function (Fig. 1, G and H).

Next, we assessed the role of cross-presenting conventional DC type 1 (cDC1s), which are essential for priming CD8^+^ T cells during RT (Vanpouille-Box et al., 2017; Böttcher and Reis e Sousa, 2018; Tadepalli et al., 2023). Depleting cDC1s did not alter tumor growth after RT (Fig. S1 E). However, cDC1 elimination in Treg-depleted mice abolished the RT-induced immune response (Fig. 1 I), suggesting that Tregs suppress cDC1 function after RT.

We concluded that Tregs mediate melanoma resistance to conformal RT by suppressing TIL and cDC1 function.

### RT promotes moDCs accumulation in radioresistant tumors

**Figure 2.**
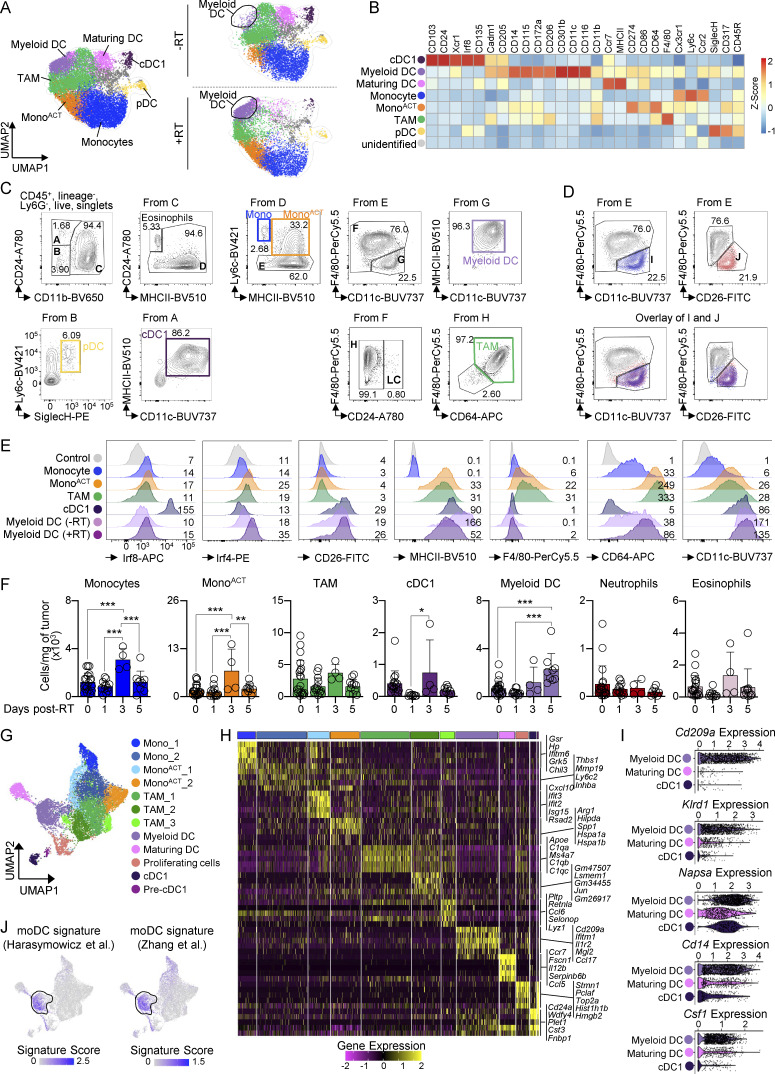
**RT induces accumulation of moDCs in the TME. (A and B)** B16-bearing B6 mice on days 0 (−RT) and 5 after RT (+RT), analyzed by CyTOF. Mononuclear phagocytes were gated as live/singlets/CD45^+^/CD3^−^/CD19^−^/CD335^−^/CD8^−^, and eosinophils/neutrophils were excluded (*n* = 2/group, 2 experiments [exp.]). **(A)** Left: UMAP generated using FlowSOM. Right: UMAPs of all cells and treatments (contour plot) were overlaid with cell populations on days 0 (−RT) and 5 after RT (+RT). **(B)** Heatmap of the Z-scored expression of markers in each cluster identified by FlowSOM. **(C and D)** B16 tumors analyzed by flow cytometry. **(C)** Representative gating strategy of mononuclear phagocytes gated on live/singlets/CD45^+^/Ly6G^−^/lineage^−^ (lineage staining includes CD3, CD19, and NK1.1). **(D)** As in C, but the expression of F4/80, CD11c, and CD26 on cells within gate E is shown. **(E)** Representative expression of canonical markers, by flow cytometry. Numbers indicate gMFI ×10^2^. Myeloid DCs are shown on days 0 (−RT) and 5 after RT (+RT). All the rest of the populations are shown on day 5 after RT (1 of 3 exp.). **(F)** Cell numbers per mg of tumor on days 1, 3, and 5 after RT, by flow cytometry (mean + SD; *n* = 4–20/group, 4–5 exp.). Statistics: one-way ANOVA plus Tukey’s post hoc test. *P ≤ 0.05, **P ≤ 0.01, ***P ≤ 0.001. **(G)** Mononuclear phagocytes were analyzed on days 0 and 5 after RT, by scRNAseq. 5,000 mononuclear phagocytes (excluding granulocytes and pDCs) were plotted. **(H)** Heatmap displays the top five DEGs for each cluster. Gene expression levels are normalized as log-transformed counts per cell for each cluster, as indicated in Table S2. **(I)** Normalized gene expression levels for the indicated genes, by scRNAseq. **(J)** moDC signatures overlaid onto the UMAP of scRNAseq data. Expression values represent log-normalized counts for genes listed in Table S1. gMFI, geometric mean fluorescence intensity.

To identify the myeloid cells responsible for Treg accumulation, we performed mass cytometry (CyTOF) (Fig. 2, A and B). Unbiased clustering identified distinct myeloid populations, including monocytes expressing Ly6C and Ccr2; mono^ACT^ expressing monocyte and activation/maturation markers (MHCII, CD86, and CD274); tumor-associated macrophages (TAMs) expressing high levels of CD64 and F4/80; cDC1s expressing Xcr1 and CD24; plasmacytoid dendritic cells (pDCs) expressing Siglec-H and CD317; maturing DCs expressing high levels of activation/migration markers such as Ccr7 and MHCII; and a distinct DC cluster expressing high levels of CD11c, CD11b, CD172a, and CD301b, resembling DC2s. To avoid nomenclature confusions before determining their origin (see below), we referred to this last population as “myeloid DCs.”

**Figure S2.**
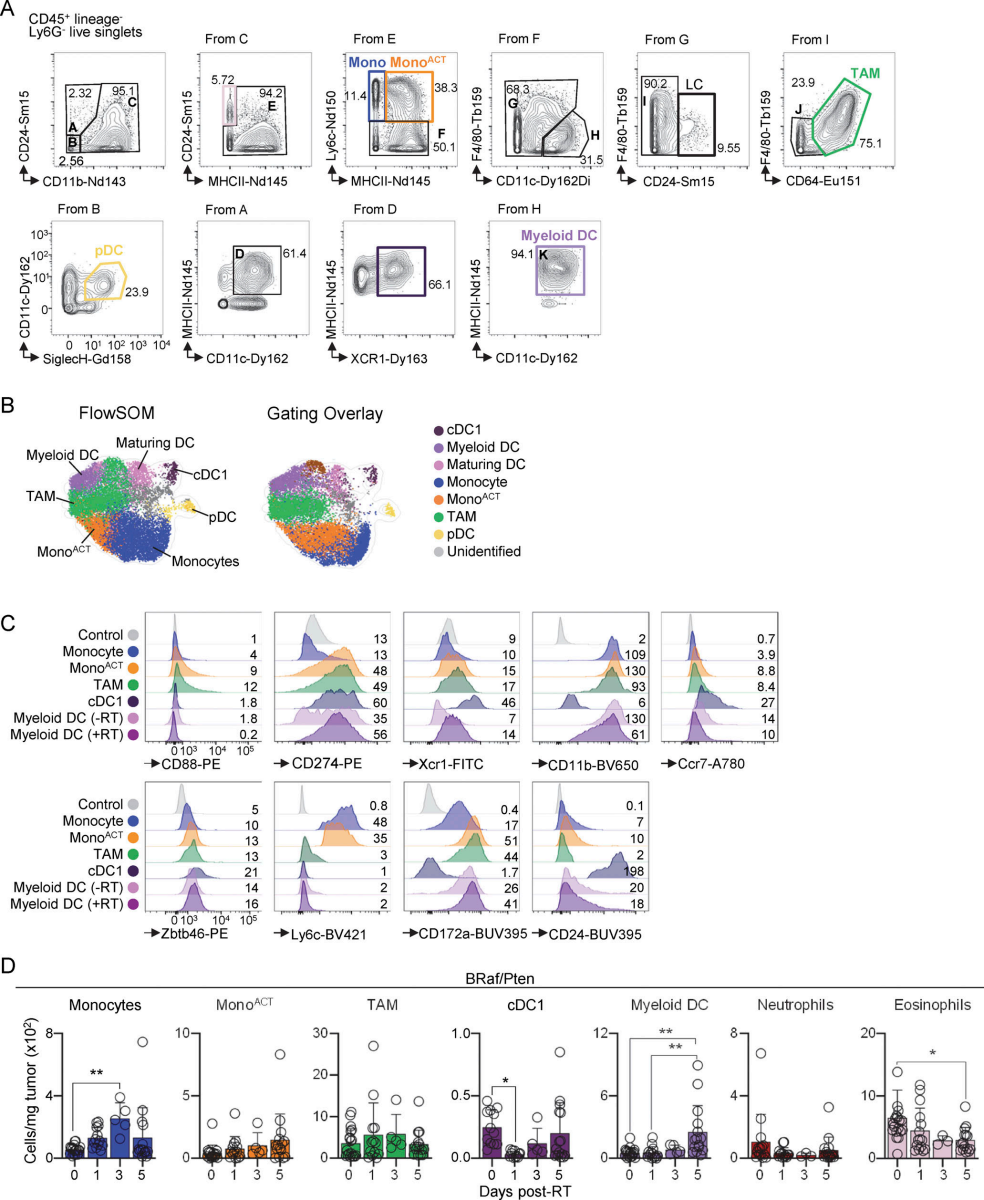
**Myeloid cell analysis after RT. (A and B)** B16 tumors analyzed by CyTOF. **(A)** Representative gating strategy of live/singlets/CD45^+^/Ly6G^−^/lineage^−^ (lineage staining includes CD3, CD19, and NK1.1). **(B)** Left: UMAP showing clusters generated using FlowSOM (Fig. 2 A). Right: cell populations identified via CyTOF gating were overlaid onto the depicted UMAP. **(C)** Representative expression of canonical markers, by flow cytometry. Numbers indicate gMFI ×10^2^. Myeloid DCs are shown on days 0 (−RT) and 5 (+RT) after RT, while all the rest of the populations are shown at 5 days after RT (*n* = 1 of 3 experiments [exp.]). **(D)** BRaf/Pten tumors analyzed on days 1, 3, and 5 after RT, by flow cytometry (mean + SD; *n* = 5–19/group, 3–5 exp.). Statistics: one-way ANOVA plus Tukey’s post hoc test (D). *P ≤ 0.05, **P ≤ 0.01. gMFI, geometric mean fluorescence intensity.

To further characterize myeloid DCs, we designed a gating strategy based on CyTOF analysis, which was corroborated by overlaying these populations onto the UMAP of all the clusters (Fig. 2 C; and Fig. S2, A and B). Using previously described DC markers (Bosteels et al., 2020; Rawat et al., 2023), we observed that myeloid DCs expressed several DC2 markers, including CD26, but not F4/80 (Fig. 2 D). They also expressed higher levels of Irf4 and CD11c, but lower levels of CD64 and F4/80 than TAMs (Fig. 2 E). Moreover, myeloid DCs expressed CD172a, but not CD88 (Fig. S2 C). Quantification of cell populations showed that tumor-infiltrating monocytes and mono^ACT^ increased in the TME at 3 days after RT, consistent with our findings in MC38 tumors (Tadepalli et al., 2023). However, unlike MC38 tumors, we observed myeloid DC accumulation at 5 days after RT in both B16 and BRaf/Pten melanoma tumors (Fig. 2, A and F; and Fig. S2 D).

**Figure S3.**
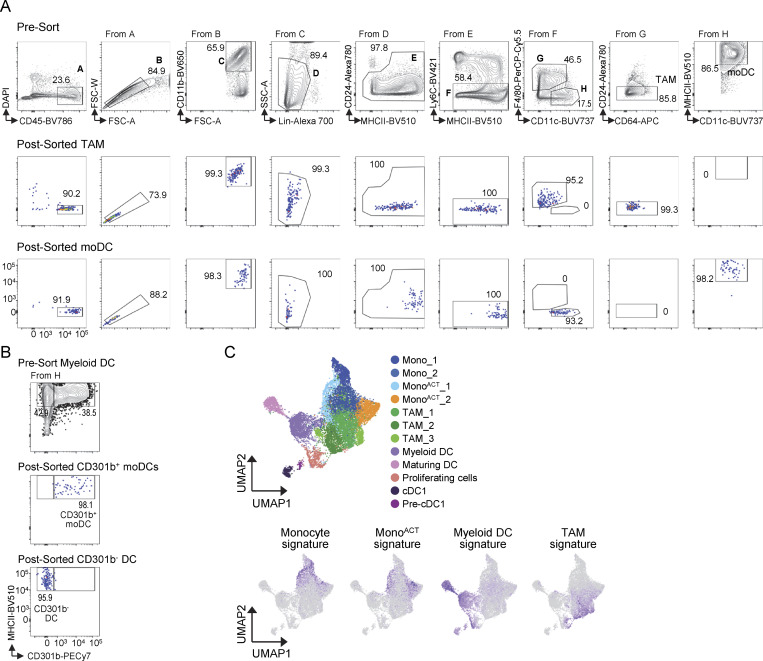
**Gating strategy and gene signature assignment. (A)** Cells were purified from B16 tumors on days 0 and 5 after RT, by FACS. Gating strategy of presorted cells (top), postsorted TAMs (middle), and postsorted DC2s/moDCs (bottom) (1 of 3 experiments [exp.]). **(B)** As in A, but myeloid DCs were sorted based on the expression of CD301b (1 of 2 exp.). **(C)** Gene signatures were overlaid onto the scRNAseq UMAP from Fig. 2 G; expression values represent log-normalized counts (genes listed in Table S1). The gene signatures of monocytes and mono^ACT^ were obtained from NanoString data as previously described (Tadepalli et al., 2023), while TAM and myeloid cell signatures were obtained from NanoString data of sorted cells, as described in A.

To further evaluate the identity of myeloid DCs, we performed single-cell RNA sequencing (scRNAseq). We analyzed 5,000 single-cell transcriptomes from nontreated and RT-treated B16 tumors using unsupervised clustering, which identified 12 distinct clusters (Fig. 2, G–I). We assigned identity to these clusters using cell-specific signatures and differentially expressed gene (DEG) analysis (Fig. S3 A for purification strategies; Fig. S3 C and Table S1 for signatures). The monocyte signature was represented in two clusters expressing high levels of *Ly6c2* and *Chil3*, an immunoregulatory monocyte marker (Baharom et al., 2022) (Fig. 2 H and Table S2). The mono^ACT^ signature was represented in two clusters: mono^ACT^_1, which expressed high levels of *Cxcl10* and interferon-stimulated genes (ISGs; *Ifit3*, *Ifit2*, *Isg15*, and *Rsad2*), and mono^ACT^_2 cluster, which expressed *Arg1* and *Hilpda*, genes associated with immunosuppression (Calcagno et al., 2020; Menjivar et al., 2023). The TAM signature labeled three clusters, all expressing macrophage-associated genes (*Apoe*, *Ms4a7*, *and C1qa/b/c*) (Zhang et al., 2020). Finally, the DC signature was represented in three clusters: one cDC1 cluster expressing *Cd24a* and *Wdfy4*, one cluster of maturing DCs expressing *Ccr7*, *Fscn1*, *Ccl5*, and *Cd274*, and one cluster expressing high levels of *Cd301b/Mgl2*, *Cd74*, and *Itgax*, likely corresponding to the myeloid DCs identified by CyTOF. This last cluster also expressed higher levels of monocyte-associated genes, i.e., *Cd209a*, *Klrd1*, *Napsa*, *Cd14*, and *Csf1* (Fig. 2 I), suggesting a monocytic origin. Indeed, applying two published moDCs signatures (Zhang et al., 2020; Harasymowicz et al., 2021) confirmed that the myeloid DC cluster was monocyte-derived (Fig. 2 J and Table S1).

Thus, resistance to RT in melanoma correlates with an increase in myeloid DCs, which appear to originate from monocytes.

### CD301b is preferentially expressed on moDCs after RT

**Figure 3.**
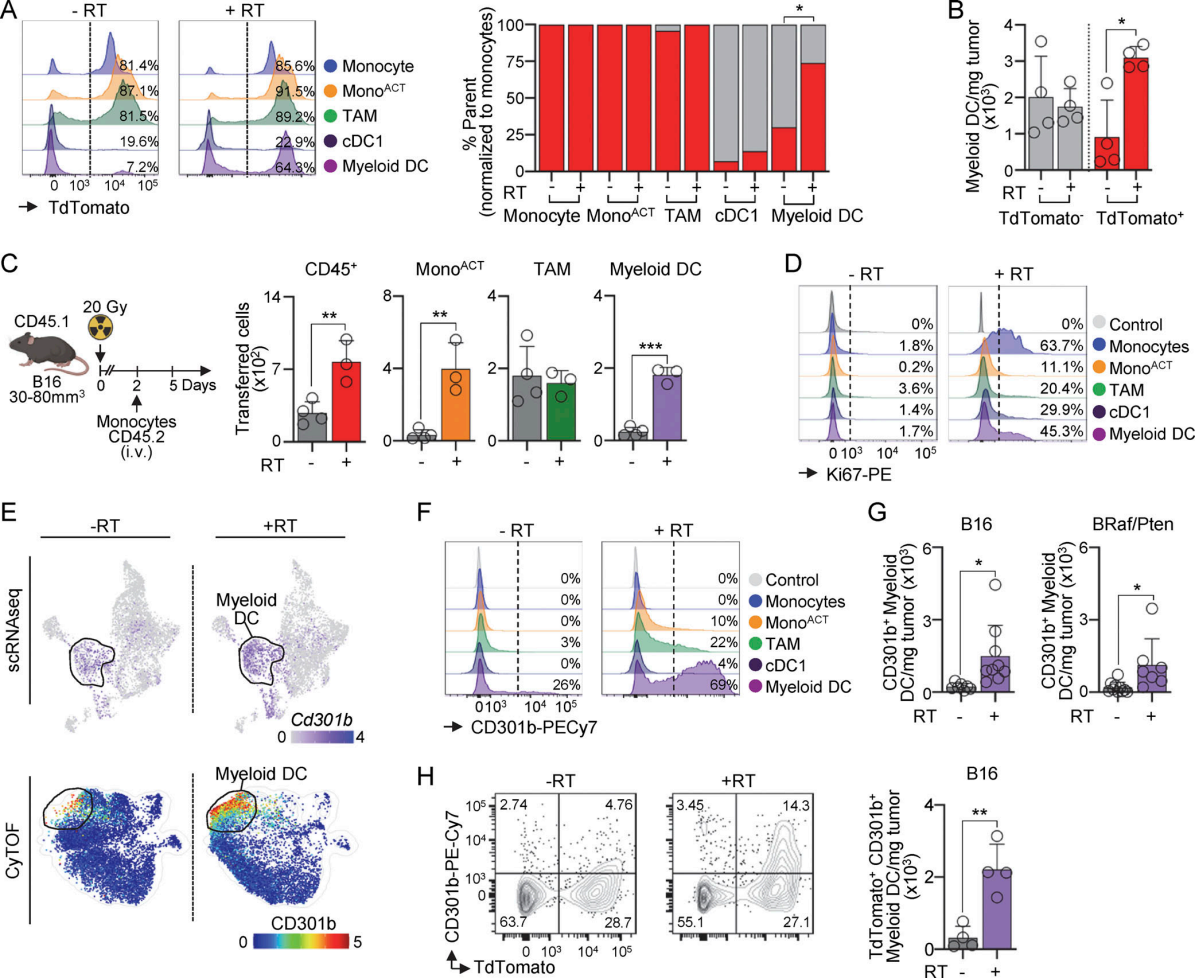
**CD301b is preferentially expressed on moDCs after RT. (A and B)** Mononuclear phagocytes in Ms4a3^*Cre*^ × Rosa^*LSL-TdTomato*^ B16–bearing mice on days 0 and 5 after RT, analyzed by flow cytometry. **(A)** Left: representative histograms of TdTomato expression. Numbers represent the frequency of cells expressing TdTomato. Right: frequency of TdTomato^+^ and TdTomato^−^ cells in *Ms4a3*^Cre^ × *Rosa*^LSL-TdTomato^ tumor–bearing mice (mean; *n* = 4/group, 2 experiments [exp.]). **(B)** Total number of TdTomato^+^ and TdTomato^−^ myeloid DCs in Ms4a3^Cre^ xRosa^LSL-TdTomato^ tumor–bearing mice (mean + SD; *n* = 4/group, 2 exp). **(C)** Bone marrow CD45.2 *Ccr2*^RFP+^*Cx3cr1*^EGFP+^ monocytes were adoptively transferred into B16-bearing CD45.1 mice 2 days after RT. Left: experimental design. Right: numbers and identity of transferred cells 5 days after RT, by flow cytometry (mean + SD; *n* = 3–4/group, 2 exp.). **(D)** Expression of Ki-67 by mononuclear phagocytes on days 0 and 5 after RT, by flow cytometry. **(E)** Top: relative expression of *Cd301b* is overlaid onto the UMAP from Fig. 2 G, by scRNAseq; expression values represent log-normalized counts. Bottom: relative expression of CD301b is displayed onto the UMAP of all populations on days 0 and 5 after RT (Fig. 2 A), by CyTOF; expression values represent an arcsinh transformation of marker intensity. **(F)** Relative expression of CD301b in B16 tumors on days 0 and 5 after RT, by flow cytometry (*n* = 1 of 2 exp.). Numbers represent the frequency of positive cells. **(G)** Number of CD301b^+^ myeloid DCs per mg of tumors in B16 (left), and BRaf/PTEN (right) on days 0 and 5 after RT, by flow cytometry (mean + SD; *n* = 7–9/group, 2–3 exp.). **(H)** Left: expression of CD301b and *Ms4a3*^Cre^ × *Rosa*^LSL-TdTomato^ on days 0 and 5 after RT, by flow cytometry. Right: number of TdTomato^+^ CD301b^+^ myeloid DCs per mg of tumor on days 0 and 5 after RT, by flow cytometry (mean + SD; *n* = 4/group, 2 exp.). Statistics: two-tailed *t* test (A–C, G, and H). *P ≤ 0.05, **P ≤ 0.01, ***P ≤ 0.001.

To evaluate experimentally whether myeloid DCs originate from monocytes, we used the *Ms4a3*^Cre^ × *Rosa*^LSL-TdTomato^ fate-tracing mouse model. These mice label monocytes and monocyte-derived cells with TdTomato, but not conventional DCs (Liu et al., 2019). Approximately 65–75% of myeloid DCs were TdTomato^+^ 5 days after RT, but not before treatment (Fig. 3 A). Cell quantification showed that only TdTomato^+^ myeloid DCs increased after RT (Fig. 3 B), suggesting that RT promotes the recruitment and/or proliferation of monocytes and their differentiation into moDCs. To test this, we performed monocyte adoptive transfer experiments. RT promoted the accumulation of transferred monocytes and their differentiation into myeloid DCs 5 days after RT (Fig. 3 C). Moreover, Ki-67 staining revealed a higher frequency of proliferating monocytes and myeloid DCs 5 days after RT (Fig. 3 D). Altogether, these data show that RT promotes monocyte infiltration into tumors, their proliferation, and differentiation into moDCs.

We noticed that *Cd301b*/CD301b expression was present in a small fraction of myeloid DCs before treatment and increased significantly after RT in our scRNAseq and CyTOF analyses (Fig. 3 E). Flow cytometry cell quantification confirmed the increase in CD301b^+^ myeloid DCs after RT (Fig. 3, F and G). We next correlated CD301b expression with the monocytic origin of myeloid DCs after RT using *Ms4a3*^Cre^ × *Rosa*^LSL-TdTomato^ mice. After RT, almost all CD301b^+^ cells expressed TdTomato, which correlated with the increase in the number of double-positive CD301b^+^ TdTomato^+^ myeloid DCs (Fig. 3 H). Notably, unlike other models (Raes et al., 2005; Kumamoto et al., 2016a; Shook et al., 2016), CD301b was mainly expressed by myeloid DCs and only minimally by TAMs (Fig. 3 F).

In summary, myeloid DCs have a phenotype very similar to DC2s (Fig. 2 E and Fig. S2 C). However, after RT, myeloid DCs display a monocytic origin and are marked by their high expression of CD301b. Altogether, our data identified myeloid DCs as DC2s before RT and as moDCs after RT.

### CD301b^+^ moDCs mediate Treg generation after RT

**Figure S4.**
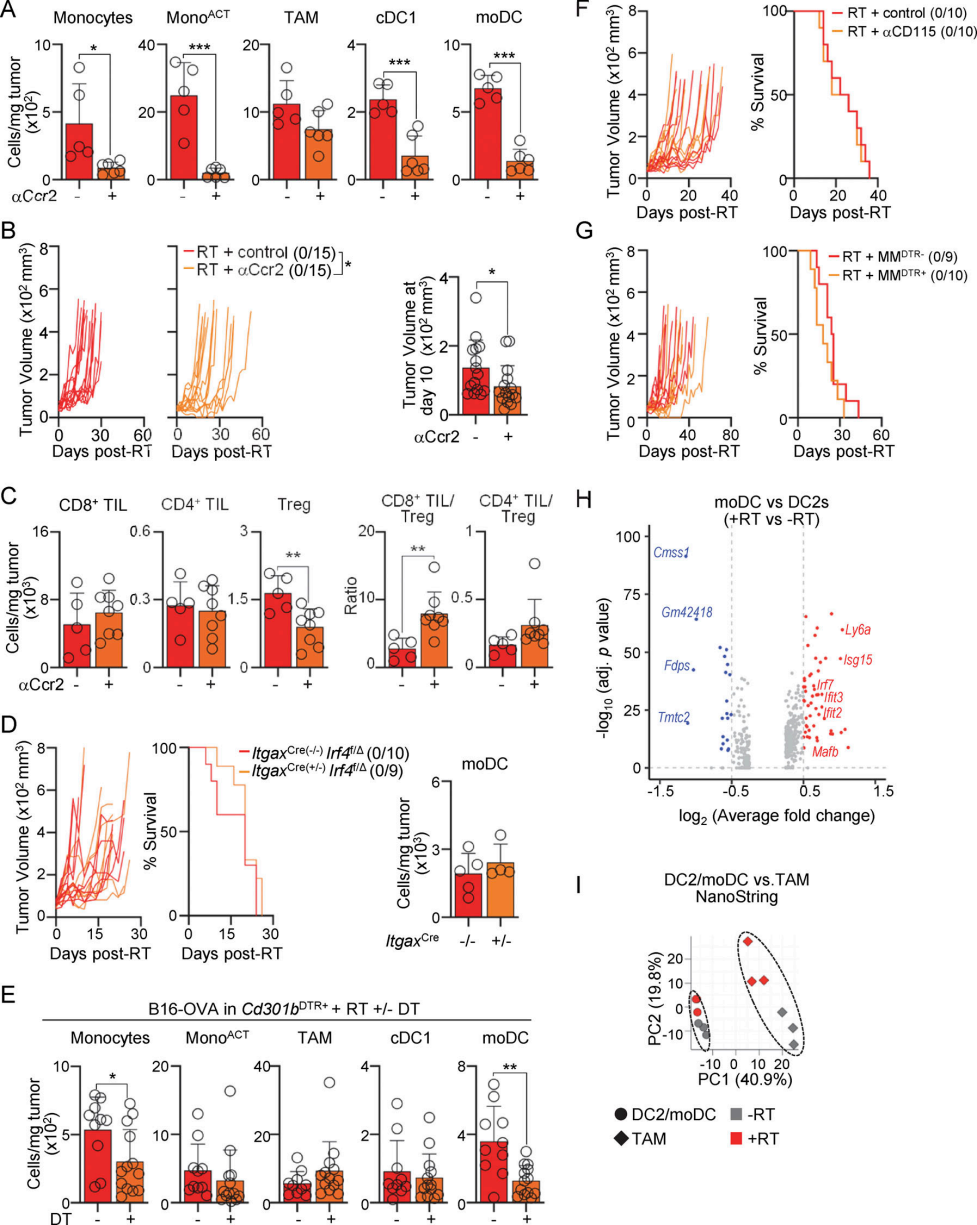
**Functional and transcriptional features of moDCs. (A–C)** B16-bearing B6 mice after RT, and αCcr2 or control Ab inoculation. **(A)** Cells per mg of tumor 5 days after RT, by flow cytometry (mean + SD; *n* = 5–6/group, 3–4 experiments [exp.]). **(B)** Left: individual tumor growth with ratio of surviving mice in parentheses. Right: tumor volume 10 days after RT (mean + SD; *n* = 15/group, 3 exp.). **(C)** TIL numbers per mg of tumor and TIL/Treg ratio 7 days after RT, by flow cytometry (mean + SD; *n* = 5–8/group, 3–4 exp.). **(D)** B16-bearing *Itgax-*Cre × *Irf4*^*f/Δ*^ mice after RT. Left: individual tumor growth and survival curves with a ratio of surviving mice in parentheses (mean + SD; *n* = 9–10/group, 2 exp). Right: number of moDCs per mg of tumor 5 days after RT analyzed, by flow cytometry (mean + SD; *n* = 4–5/group, two to three exp). **(E)** B16-OVA–bearing *Cd301b*^DTR+^ after RT, with or without DT inoculation. Cells per mg of tumor 5 days after RT, by flow cytometry (mean + SD; *n* = 10–13/group, 4–7 exp.). **(F)** B16-bearing B6 mice after RT, and αCD115 or control Ab inoculation. Individual tumor growth and survival curves with a ratio of surviving mice in parentheses (*n* = 10/group, 2 exp.). **(G)** B16-bearing MM^DTR^ mice after RT and DT inoculation. Individual tumor growth and survival curves with a ratio of surviving mice in parentheses (*n* = 9–10/group, 2 exp.). **(H)** DEGs between DC2s (day 0; −RT) and moDCs (day 5 after RT; +RT) are shown using a log_2_ fold change cutoff of 0.5, by scRNAseq. **(I)** NanoString transcriptomic analysis of sorted DC2s/moDCs versus TAMs on days 0 (−RT) and 5 after RT (+RT). PCA of normalized sample counts is shown. Statistics: two-way ANOVA plus Tukey’s post hoc test for mean tumor growth (B, D, F, and G); Mantel–Cox’s test for survival curves (D, F, and G); two-tailed *t* test (A–E). *P ≤ 0.05, **P ≤ 0.01, ***P ≤ 0.001.

Our data indicate that RT resistance is caused by the accumulation of Tregs. To assess the role of moDCs in Treg generation and RT resistance, we depleted them using antibodies (Abs) against Ccr2 (αCcr2), a chemokine receptor required for monocyte mobilization outside the bone marrow (Hohl et al., 2009). αCcr2 Abs resulted in the depletion of monocytes, mono^ACT^, and moDCs, but interestingly not TAM after RT (Fig. S4 A). It also resulted in the depletion of cDC1 in this model, which was attributed to Ccr2 expression in cDC1 precursors or pre-cDC1s (Pereira da Costa et al., 2023). Nevertheless, αCcr2 Ab inoculation decreased Tregs and increased treatment efficacy (Fig. S4, B and C), suggesting a role of monocytes and monocyte-derived cells in RT resistance.

**Figure 4.**
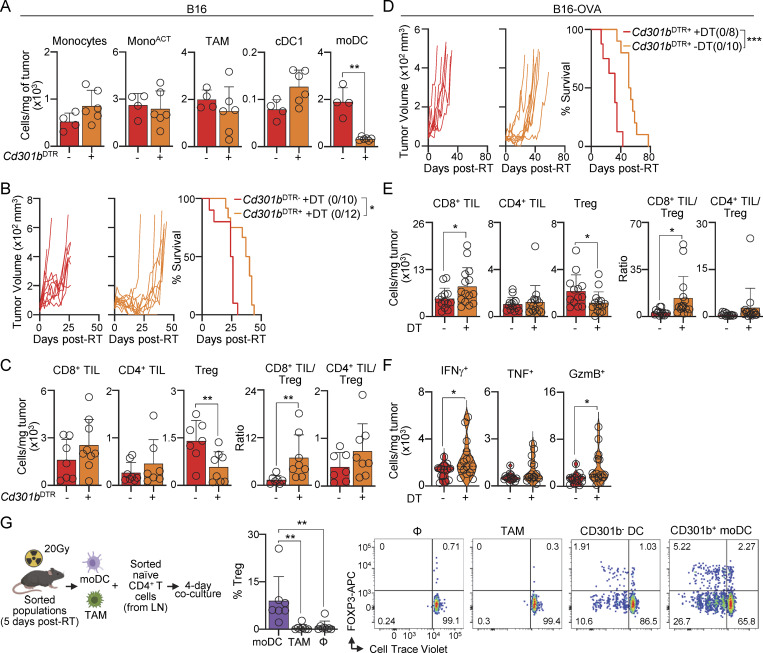
**CD301b** ^**+**^ **moDCs mediate Treg generation after RT. (A–C)** B16-bearing *Cd301b*^DTR+^ and *Cd301b*^DTR−^ mice after RT and DT inoculation. **(A)** Cells per mg of tumor 5 days after RT, by flow cytometry (mean + SD; *n* = 4–6/group, 2 experiments [exp.]). **(B)** Individual tumor growth and survival curves with a ratio of surviving mice in parentheses (*n* = 10–12/group, 2 exp.). **(C)** TIL numbers and TIL-to-Treg ratio on days 0 and 7 after RT, by flow cytometry (mean + SD; *n* = 7–9/group, 3 exp.). **(D–F)** B16-OVA–bearing *Cd301b*^DTR+^ after RT, with or without DT inoculation. **(D)** Individual tumor growth and survival curves with a ratio of surviving mice in parentheses (*n* = 8–10/group, 2 exp.). **(E)** TIL numbers and TIL-to-Treg ratio on days 0 and 7 after RT, by flow cytometry (mean + SD; *n* = 13–17/group, 4–6 exp.). **(F)** TILs producing IFNγ, TNF, and GzmB 7 days after RT, after ex vivo restimulation, by flow cytometry (violin plots show data distribution; *n* = 13–17/group, 4–6 exp.). **(G)** Tumor-infiltrating moDCs and TAMs were sorted (5 days after RT; Fig. S3, A and B, for purification strategies) and co-cultured with naïve CD4^+^ T cells purified from LNs of B6 mice. Analysis was done 4 days later. Left: experimental schematic. Middle: frequency of Treg within CD4^+^ T cells (mean + SD; *n* = 5–8/group, five to seven exp). Right: representative flow cytometry plot showing Treg generation by sorted CD301b^+^ moDCs versus CD301b^−^ myeloid DCs and TAMs (*n* = 1 of 2 exp.). Statistics: two-way ANOVA plus Tukey’s post hoc test for mean tumor growth (B and D); Mantel–Cox’s test for survival curves (B and D); one-way ANOVA plus Tukey’s post hoc test (G); two-tailed *t* test (A, C, E, and F). *P ≤ 0.05, **P ≤ 0.01, ***P ≤ 0.001.

To specifically deplete moDCs, we first tested *Itgax-*Cre x*Irf4*^*f/∆*^ mice (Gao et al., 2013) since these cells express Irf4 (Fig. 2 E). However, these mice had normal numbers of moDCs after RT and no change in treatment efficacy (Fig. S4 D). Next, we used *Cd301b*^*DTR*^ mice in which DT inoculation resulted in the specific depletion of CD301b-expressing moDCs (Fig. 4 A). MoDC depletion resulted in a significant delay in tumor growth and prolonged survival in comparison with control mice (Fig. 4 B), which correlated with decreased Treg numbers and an increased CD8^+^ TIL-to-Treg ratio after treatment (Fig. 4 C). The same results were observed in B16 tumors expressing the neoantigen ovalbumin (B16-OVA); i.e., specific depletion of CD301b-expressing moDCs improved RT efficacy, decreased Treg numbers, and increased CD8^+^ T cell numbers and function (Fig. 4, D–F and Fig. S4 E). Notably, depletion of TAMs using two different mouse models, i.e., αCD115 Abs and MM^DTR^ mice (Schreiber et al., 2013), did not result in an increase in survival after RT (Fig. S4, F and G).

Next, we evaluated whether moDCs have an intrinsic capacity to generate Treg (Fig. 4 G). Naïve CD44^−^CD45RB^+^CD4^+^ T cells obtained from lymph nodes (LNs) of tumor-free B6 mice were co-cultured with tumor-infiltrating TAMs or moDCs purified 5 days after RT (Fig. S3 A for purification strategies). The frequency of FOXP3^+^ Tregs was significantly higher when naïve CD4^+^ T cells were co-cultured with moDCs (Fig. 4 G). Moreover, moDCs expressing CD301b have a superior capacity to promote Tregs compared with myeloid DCs lacking CD301b expression (Fig. 4 G and Fig. S3 B for purification strategies). Together, our data support a model in which RT promotes the accumulation of CD301b^+^ moDCs in radioresistant tumors, which results in Treg generation and the consequent low efficacy of this treatment.

### MoDCs are transcriptionally similar to DC2 and differ from TAMs

**Figure 5.**
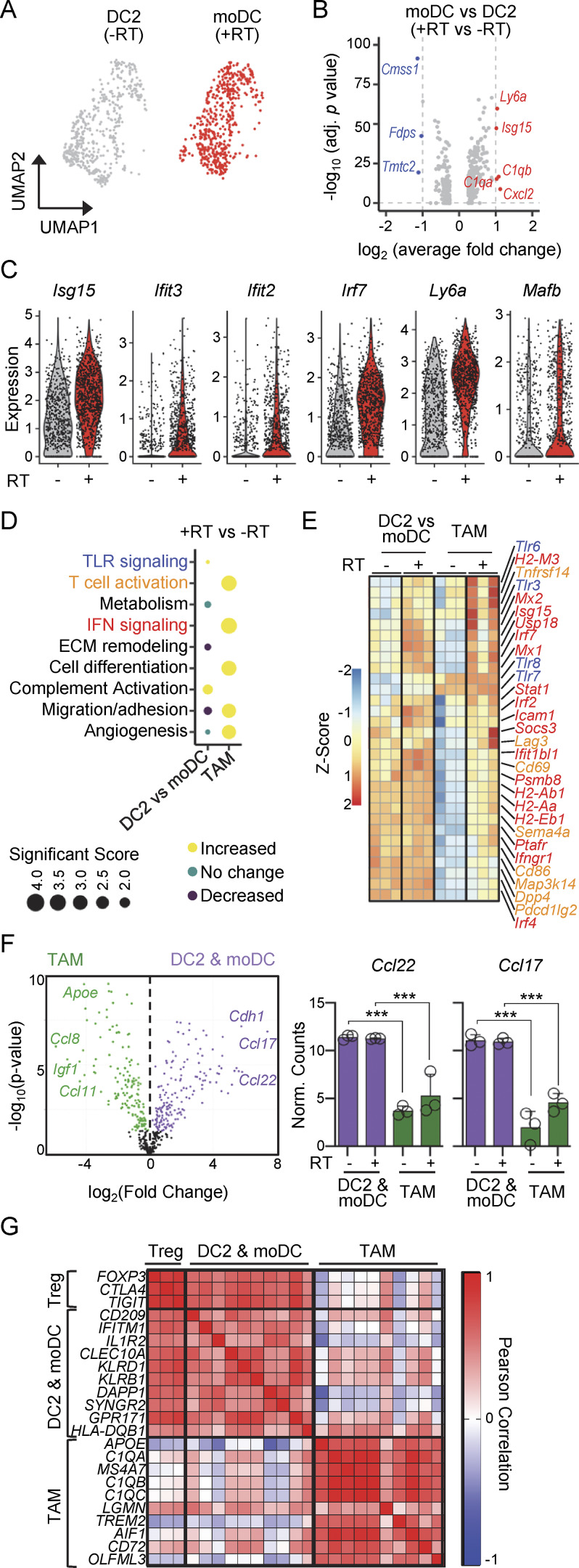
**moDCs are transcriptionally similar to DC2s and differ from TAMs. (A)** UMAP of DC2s and moDCs on days 0 and 5 after RT, respectively, by scRNAseq. **(B)** DEGs between DC2s (day 0) and moDCs (day 5 after RT) are shown using a log_2_ fold change threshold of 1, by scRNAseq. **(C)** Expression of the indicated genes in DC2s (day 0) and moDCs (day 5 after RT), by scRNAseq. **(D–F)** NanoString transcriptomic analysis of sorted DC2s/moDCs and TAMs before (0 days) and after (5 days) RT (mean + SD; *n* = 3/group in 3–4 experiments [exp.]). **(D)** Pathway enrichment analysis was performed on normalized counts. The size indicates the significance of the score. **(E)** Heatmap of normalized counts comparing DC2s/moDCs with TAMs 0 versus 5 days after RT, by NanoString (see Table S4). **(F)** Left: DEGs significantly upregulated in DC2s/moDCs relative to TAMs. Right: normalized expression counts for the indicated genes, stratified by cell type and treatment. Statistics: one-way ANOVA plus Tukey’s post hoc test. ***P ≤ 0.001. **(G)** Pearson’s correlation matrix of indicated genes in patients undergoing RT, derived from The Cancer Genome Atlas for radioresistant tumors (melanoma, glioblastoma, and head and neck cancer).

Less than 25% of cells called myeloid DCs traced to monocytes before RT, demonstrating that these are not monocyte-derived and probably correspond to DC2s based on their phenotype (e.g., high levels of CD11c, CD172a, MHCII, and CD26). After RT, however, ∼65–75% of myeloid DCs are moDCs. With the goal of identifying differences between these cells, we performed single-cell transcriptomic analysis. Interestingly, the transcriptomes of DC2s (before RT) and moDCs (after RT) were almost indistinguishable (Fig. 5 A). Indeed, <10 genes were differentially expressed between DC2s/moDCs when considering a log_2_ change ≥1, and ∼50 genes when considering a log_2_ change ≥0.5 (Fig. 5 B, Fig. S4 H, and Table S3). Among genes that underwent a log_2_ change ≥0.5 following RT, we observed monocyte-associated genes such as *Ly6a*, which is consistent with a shift from a nonmonocytic to a monocytic origin for these cells (Fig. 5 C). Additionally, RT promoted slight upregulation of ISGs such as *Isg15*, *Ifit3*, *Ifit2*, and *Irf7*. We concluded that the transcriptome of moDCs converged with that of DC2s after RT of melanoma.

TAMs have been associated with Treg accumulation following shielded RT (Xu et al., 2013; Deng et al., 2014a). Thus, we next compared changes in TAMs versus DC2s/moDCs using NanoString. TAMs underwent robust changes after RT including the upregulation of T cell activation pathways, IFN signaling pathways, and cell differentiation pathways (Fig. 5, D and E; Fig. S4 I; and Table S4). In contrast, DC2/moDC gene expression barely changed after RT, in agreement with the scRNAseq data, suggesting that these cells generate Tregs independently of the treatment. Indeed, DC2s/moDCs expressed higher levels of *Ccl22* and *Ccl17*, chemokines involved in Treg generation (Mizukami et al., 2008; Rapp et al., 2019; Watanabe et al., 2019), irrespective of treatment (Fig. 5 F).

We then asked whether the gene signature of DC2s/moDCs correlates with a Treg signature in patients. Human DC2/moDC and TAM signatures were generated based on our scRNAseq (Fig. 5 G) and correlated with three Treg-associated genes (i.e., *FOXP3*, *CTLA4*, and *TIGIT*) in patients undergoing RT of radioresistant tumors (i.e., melanoma, glioblastoma, and head and neck cancer). The DC2/moDC signature, but not the TAM signature, had a positive Pearson correlation coefficient with Treg genes (Fig. 5 G).

Altogether, our results point to a model in which resistance of melanoma to RT is mediated by the number of moDCs after treatment, and not their distinct developmental origin.

### Tumor-derived GM-CSF drives the accumulation of moDCs

**Figure S5.**
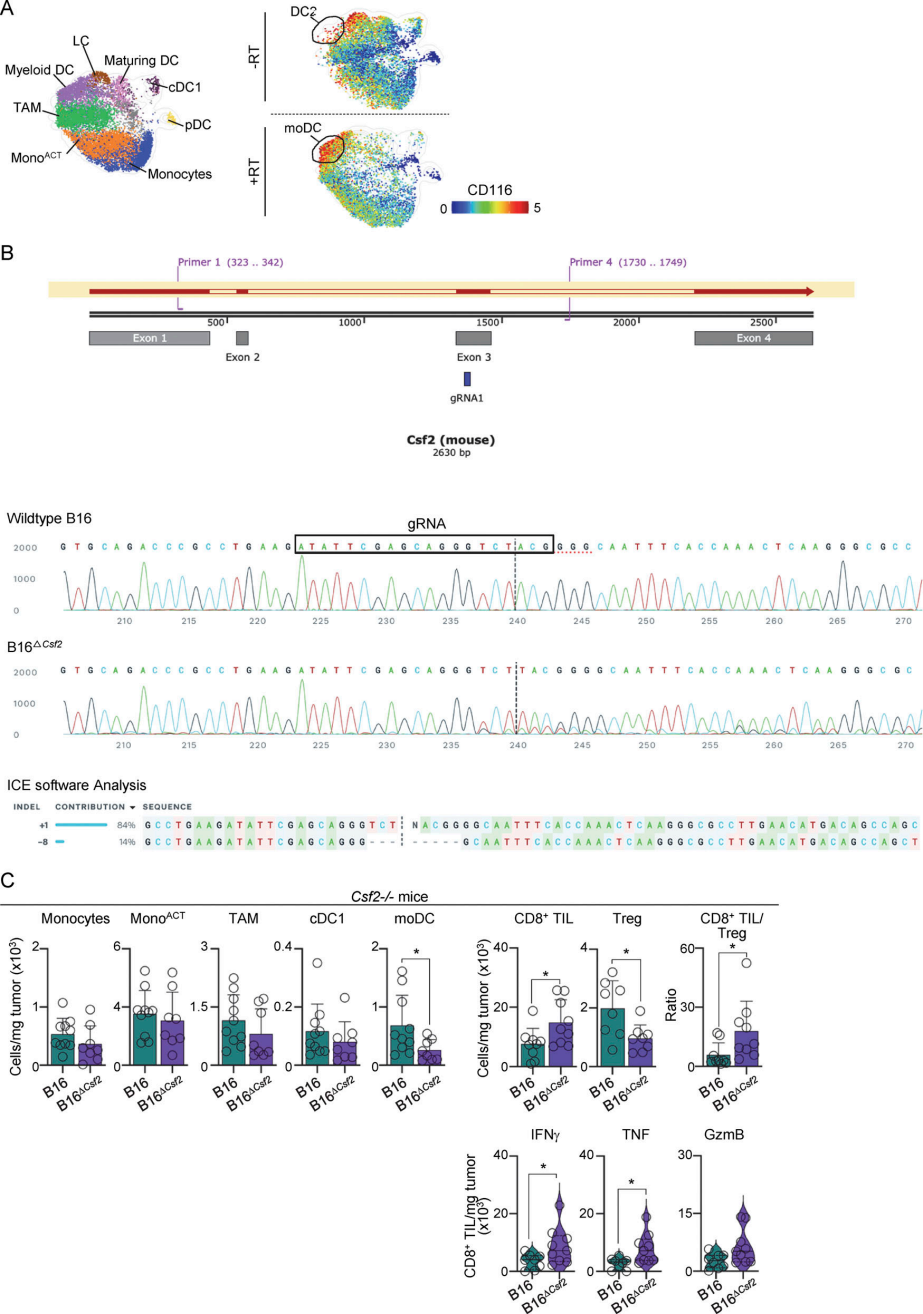
**Role of GM-CSF on CD301b** ^**+**^ **moDCs. (A)** B16 tumors analyzed by CyTOF. Left: UMAP showing clusters generated using FlowSOM (Fig. 2 A). Right: relative expression of CD116 on days 0 (−RT) and 5 after RT (+RT). Expression values represent an arcsinh transformation. **(B)** Generation of the B16^*∆Csf2*^ cell line. The line above indicates the gRNA position. ICE software analysis of Sanger sequencing data confirmed a deletion in the B16^*∆Csf2*^ cell clone (below). **(C)***Csf2*^−/−^ mice transplanted with B16 and B16^*∆Csf2*^ tumor cells. Left: cells per mg of tumor at 5 days after RT, by flow cytometry (mean + SD; *n* = 8–9/group, 4–5 experiments [exp.]). Right: TIL numbers, TIL/Treg ratio, and CD8^+^ TILs producing IFNγ, TNF, and GzmB at 7 days after RT, after ex vivo restimulation, by flow cytometry (mean + SD and violin plots of data distribution; *n* = 8–11/3–5 exp.). Statistics: two-tailed *t* test. *P ≤ 0.05.

**Figure 6.**
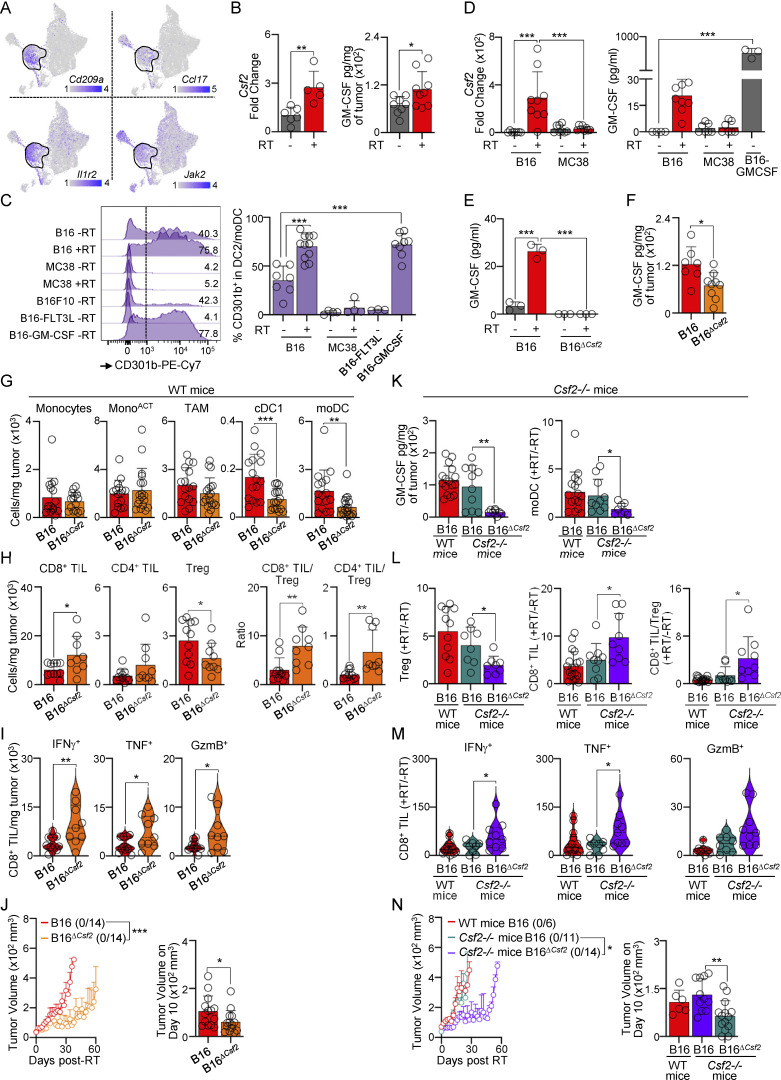
**Tumor-derived GM-CSF drives the accumulation of CD301b** ^**+**^ **moDCs. (A)** Relative expression of selected genes was overlaid onto the UMAP from Fig. 2 G; expression values represent log-normalized counts. **(B)** B16-bearing mice on days 0 and 5 after RT. Left: tumor *Csf2* transcript levels, by qPCR (mean + SD; *n* = 5–6/group, 2–4 experiments [exp.]). Right: GM-CSF protein levels in tumor lysate, by ELISA (mean + SD; *n* = 6–9/group, 4 exp.). **(C)** Expression of CD301b by DC2s/moDCs, by flow cytometry. Left: frequency of CD301b on DC2s (day 0) and moDCs (day 5 after RT) (1 of 3 exp.). Right: frequency of CD301b^+^ expressing cells (mean + SD; *n* = 3–11/group, 2–5 exp.). **(D)** B16 and MC38 tumor cells were irradiated with 20 Gy RT and cultured for 4 days. Left: tumor cell *Csf2* transcript levels, by qPCR (mean + SD; *n* = 7–9/group, 3–4 exp.). Right: GM-CSF protein levels in the culture supernatant, by ELISA (mean + SD; *n* = 4–9/group, 3–4 exp.). **(E)** B16 and B16^*∆Csf2*^ tumor cell lines were irradiated with 20 Gy and cultured for 4 days. GM-CSF protein levels in the culture supernatant (mean + SD; *n* = 3/group, 3 exp.). **(F–J)** B16 and B16^*∆Csf2*^ tumor–bearing mice treated with 20 Gy RT. **(F)** GM-CSF protein level in tumor lysate 5 days after RT, by ELISA (mean + SD; *n* = 7–9/group, four exp). **(G)** Cells per mg of tumor 5 days after RT, by flow cytometry (mean + SD; *n* = 6–22/group, 4–5 exp). **(H)** TIL numbers and TIL/Treg ratio 7 days after RT, by flow cytometry (mean + SD; *n* = 9–11/group, 4 exp). **(I)** TILs producing IFNγ, TNF, and GzmB 7 days after RT, after ex vivo restimulation, by flow cytometry (violin plots show data distribution; *n* = 9–11/group, 4 exp.). **(J)** Left: average tumor growth with a ratio of surviving mice in parentheses (mean + SEM). Right: tumor volume day 10 after RT (mean + SD; *n* = 14/group, 3 exp.). **(K–N)** As in F–J, but *Csf2*^−/−^ mice were transplanted with B16 and B16^*∆Csf2*^ tumor cells. **(K)** Left: GM-CSF protein level in tumor lysate 5 days after RT, by ELISA (mean + SD; *n* = 8–16/group, 4–5 exp.). Right: moDC numbers 5 days after RT (+RT) normalized to day 0 (−RT), by flow cytometry (mean + SD; *n* = 8–9/group, 4–5 exp.). **(L)** Cells per mg of tumor and CD8^+^ TIL-to-Treg ratio 7 days after RT, normalized to day 0 (−RT) (mean + SD; *n* = 8–11/group, 4–5 exp.). **(M)** TILs producing IFNγ, TNF, and GzmB 7 days after RT, after ex vivo restimulation, normalized to day 0 (−RT) (violin plots show data distribution, *n* = 8–9/group, three to five exp). **(N)** Left: average tumor growth with a ratio of surviving mice in parentheses (mean + SEM). Right: tumor volume 10 days after RT (mean + SD; *n* = 6–14/group, 2–3 exp). Statistics: two-way ANOVA plus Tukey’s post hoc test for mean tumor growth (J and N); one-way ANOVA plus Tukey’s post hoc test (C–E); two-tailed *t* test for the rest. *P ≤ 0.05, **P ≤ 0.01, ***P ≤ 0.001.

GM-CSF is a growth factor instrumental in the differentiation of monocytes into DCs (Greter et al., 2012; Helft et al., 2015) and has been associated with CD301b expression in DCs (Kenkel et al., 2017). Our CyTOF analysis revealed the expression of the GM-CSF receptor (CD116) on moDCs (Fig. S5 A). Furthermore, scRNAseq data showed that *Cd301b*-expressing cells have high levels of several GM-CSF–associated genes (i.e., *Cd209a*, *Ccl17*, *Il1r2*, *Jak2*) (Fig. 6 A). Based on this, we investigated whether RT promotes GM-CSF secretion in the TME. We detected higher levels of *Csf2* transcripts and GM-CSF protein in the TME 5 days after RT of B16 melanoma (Fig. 6 B). Higher GM-CSF levels after RT correlated with higher frequency of CD301b^+^ moDCs (Fig. 6 C). Moreover, accumulation of CD301b^+^ moDCs in the TME was observed in transplanted B16 tumors overexpressing GM-CSF, but not in those overexpressing FLT3L (Fig. 6 C). We then investigated whether tumor cells intrinsically produced GM-CSF. Irradiation of radioresistant B16 tumor cells in vitro, but not MC38 tumor cells, resulted in increased levels of *Csf2* transcripts and GM-CSF protein in the culture supernatant (Fig. 6 D). We concluded that B16 tumor cells produce GM-CSF following irradiation.

We generated a B16 cell line that cannot produce GM-CSF (B16^*∆Csf2*^) using CRISPR (Fig. S5 B). B16^*∆Csf2*^ cells did not secrete GM-CSF after irradiation in vitro (Fig. 6 E). B16^*∆Csf2*^ transplanted tumors produced significantly less GM-CSF in vivo after RT (Fig. 6 F), which correlated with a decrease in the number of moDCs (Fig. 6 G). B16^*∆Csf2*^ tumors responded significantly better to RT, had a decreased number of Treg, an increased TIL/Treg ratio, and an improved function of CD8^+^ TILs (Fig. 6, H–J).

To evaluate the role of non–tumor-derived GM-CSF after RT, we used *Csf2*^*−/−*^ mice. We observed no significant differences in the numbers of moDCs or Tregs, or treatment efficacy between B16 tumors transplanted into wild-type (WT) or *Csf2*^*−/−*^ mice (Fig. 6, K–N). However, similar to our results in WT mice, B16^*∆Csf2*^ tumors transplanted into in *Csf2*^*−/−*^ mice had a decrease in the number of moDCs and Tregs, and a significant improvement in treatment efficacy correlating with low GM-CSF levels (Fig. 6, K–N and Fig. S5 C for total numbers).

Altogether, our data suggest that tumor-derived GM-CSF limits the efficacy of RT in melanoma by promoting the accumulation of CD301b^+^ moDCs.

## Discussion

Our data suggest a model in which CD301b^+^ moDCs limit the therapeutic efficacy of clinically relevant conformal RT by promoting Treg generation. The accumulation of CD301b^+^ moDCs in the TME after RT appears to be driven by tumor-derived GM-CSF. These findings support the potential of targeting tumor-derived GM-CSF to enhance RT efficacy.

Classification of discrete mononuclear phagocyte lineages has allowed the field to correlate findings between mouse models and translate results to humans. These refined classifications (using high-dimensional single-cell phenotypic and transcriptional approaches combined with fate-tracing experiments) move away from all-encompassing nomenclatures such as MDSCs (Hegde et al., 2021). In this manuscript, high-dimensional approaches revealed surprising insights into tumor-infiltrating monocyte-derived cells. We observed that surface markers commonly used to identify DC2s (such as CD11c, MHCII, CD172a, and CD26) did not clearly distinguish DC2s from moDCs in RT-treated tumors. Indeed, a fate-tracing model demonstrated that over 75% of cells expressing these markers after RT are derived from monocytes. Moreover, adoptive transfer experiments confirmed that these observations are not artifacts of the tracing model. Together, our findings indicate that the microenvironment, not solely developmental origin, determines the features and function of monocyte-derived cells. Accordingly, we observed that tumor DC2s and moDCs share a similar transcriptome, including the expression of Treg-recruiting genes such as *Ccl22* and *Ccl17*. Although monocytes do not always acquire a DC2-like profile (Plantinga et al., 2013; Olingy et al., 2019; Bosteels et al., 2020; Tewari et al., 2021; Rawat et al., 2023), our results highlight their capacity to adopt similar phenotypic and functional characteristics in the TME during RT.

We show that moDCs can be identified by CD301b expression after RT. CD301b^+^ DCs have been previously reported to induce diverse immune responses, including Th2 polarization during helminth infection, allergen challenge, and immunization with blood-borne antigens (Kumamoto et al., 2013; Perner et al., 2020; Ryu et al., 2022; Lyons-Cohen et al., 2024); Th17 polarization in psoriasis (Kim et al., 2018); and the generation of tissue-resident memory CD8^+^ T cells against HSV-2 (Shin et al., 2016). Additionally, CD301b^+^ DCs can suppress immune responses by promoting Treg in models of pancreatic cancer and lung allergy (Kenkel et al., 2017; Wilkinson et al., 2024, *Preprint*), and they are also capable of inhibiting T follicular helper cells (Kumamoto et al., 2016b). These diverse functions suggest that CD301b expression may reflect DCs’ capacity to sense GM-CSF rather than marking a discrete subset (Kim et al., 2018; Ryu et al., 2022; Wilkinson et al., 2024, *Preprint*). Notably, in our model, CD301b^+^ moDCs also express *Cd209a* transcripts, suggesting they may arise from monocyte–DC progenitors, as described by Yáñez et al. (2017). Importantly, the labeling of moDCs in the *Ms4a3*^Cre^ fate-tracing model indicates that these cells are not DC type 3, a pro-inflammatory cell population with overlapping monocyte–DC features that does not trace to monocytes (Liu et al., 2023). Interestingly, while CD301b has been associated with TAMs in other models (Raes et al., 2005; Kumamoto et al., 2016a; Shook et al., 2016), we found that CD301b was not expressed by TAMs in our system, likely due to their low expression of the GM-CSF receptor (CD116) after RT. The specific expression of CD301b by moDCs enabled us to temporally deplete these cells using a previously described mouse model (Kumamoto et al., 2013) and compare their function with that of TAM after RT. Even though TAMs are known to exert an immunosuppressive role in mice and humans (Bill et al., 2023; Lynch et al., 2024), we found that CD301b^+^ moDCs also contribute to Treg accumulation in radioresistant melanoma after RT.

Our data support the idea that accumulation of immunosuppressive moDCs during RT is orchestrated by GM-CSF. This is in agreement with reports that GM-CSF promotes the accumulation of MDSCs (Filipazzi et al., 2007; Kohanbash et al., 2013; Thorn et al., 2016), which suppress immune responses through different mechanisms including IL-4R expression (Kohanbash et al., 2013), inhibition of antigen-specific CD8^+^ T cells (Bayne et al., 2012), and upregulation of prostaglandin E2 (Kalinski, 2012; Veglia et al., 2019). Notably, tumor-secreted GM-CSF during RT has also been shown to promote protumor, cell-intrinsic effects, such as increased circulating tumor cells and metastases (Vilalta et al., 2014, 2018). Here, we identify another pathogenic role of GM-CSF during RT: its ability to drive monocyte recruitment and differentiation into immunoregulatory moDCs.

However, GM-CSF has also been described to have antitumor properties by promoting DC activation and maturation (Urdinguio et al., 2013; Nebiker et al., 2014; Ushach and Zlotnik, 2016). Consequently, clinical trials have investigated its therapeutic potential in antitumor vaccination protocols (Simons et al., 1998, 1999; Soiffer et al., 1998; Luiten et al., 2005). A seminal clinical trial combining GM-CSF with RT showed that this approach promotes an abscopal effect, i.e., tumor regression at sites distant from the irradiated field (Golden et al., 2015). More recent trials combining RT, GM-CSF, and αPD-1 also reported an abscopal effect in ∼30% of patients (Xing et al., 2023; Ni et al., 2024). However, none of these trials included a control arm with patients receiving RT without GM-CSF, limiting our understanding of GM-CSF’s role during RT (Leary et al., 2019; Benna et al., 2020). Overall, GM-CSF appears to act as a double-edged sword role in cancer, with its therapeutic efficacy likely dependent on dosage, timing, and tumor type (Kumar et al., 2022). In the context of RT, controlled clinical trials are essential to narrow the gap between mechanistic preclinical studies and clinical therapeutics (Brix et al., 2017).

While our depletion data and in vitro experiments strongly support a role of CD301b^+^ moDCs in Treg generation, some uncertainties remain. One limitation is that due to their low numbers, we were unable to purify and reintroduce moDCs into mice lacking CD301b^+^ cells to directly assess whether restoring these cells would increase Treg numbers. Additionally, although our data indicate that CD301b^+^ moDCs promote Treg accumulation, we did not determine whether they induce de novo Tregs from naïve T cells or expand preexisting tumor-localized Tregs. Furthermore, the mechanisms driving Treg accumulation by moDCs remain unexplored. Our data suggest that tumor-derived GM-CSF, triggered by RT, plays a key role in CD301b^+^ moDC accumulation. However, a limitation of our study is that we did not control the timing of GM-CSF elimination. It is possible that GM-CSF has an immunostimulatory role early in tumor development and shifts to an immunoregulatory role only after RT. Understanding the timing and magnitude of GM-CSF’s effects on pro- and antitumor immune responses is crucial for developing strategies targeting this pathway effectively.

In summary, our data suggest that one mechanism of radiation resistance in melanoma is the capacity of tumor cells to secrete GM-CSF, which triggers monocyte accumulation in the TME and their differentiation into moDCs. In this system, moDCs are superior to TAMs in generating Tregs, the key effector/suppressive cells that limit treatment efficacy. This immunosuppressive mechanism not only deepens our understanding of radiation resistance but also provides valuable insights for developing therapies that target monocyte recruitment and differentiation.

## Materials and methods

### Mice and in vivo studies

WT C57BL/6J (B6 mice; Jax#000664), BRaf/Pten (Jax#013590), *Itgax-*Cre (Jax#008068), *Irf4*^f/f^ (Jax#009380), *Irf8*^f/f^ (Jax#014175), Ai9(RCL-tdT) (*Rosa*^LSL-TdTomato^; Jax#007905), *Irf8*^Δ32^ (Jax#032744), *Ms4a3*^Cre^ (Jax#036382), *Lyz2*^Cre^ (Jax#004781), *Csf1r*^LSL-DTR^ (Jax#024046), *Ccr2*^RFP^ x*Cx3cr1*^EGFP^ (Jax#032127), and *Cd301b*^DTR^ (Jax#023822) mice were obtained from Jackson Laboratory. B6-Ly5.1/Cr (CD45.1, Cat#564) mice were obtained from Charles River. *Ms4a3*^Cre^ × *Rosa*^LSL-TdTomato^ (Liu et al., 2019), *Itgax-*Cre × *Irf8*^f/f^ (Sichien et al., 2016), *Itgax-*Cre × *Irf4*^f/Δ^ (Gao et al., 2013), *Lyz2*^Cre^ × *Csf1r*^LSL-DTR^ (MM^DTR^) (Schreiber et al., 2013), *Foxp3*^DTR^ (Fontenot et al., 2003), and *Foxp3*^DTR^ × *Irf8*^Δ32^ (used in this study) were obtained through in-house breeding. Mice aged 6–8 wk were employed and kept in specific pathogen–free conditions. Both male and female mice were used, and animals were randomly assigned to experimental groups. Our humane euthanasia criteria were as follows: when tumor-bearing mice experienced a weight loss exceeding 10%, when the tumor size reached 1.3 cm in diameter, or if tumors exhibited signs of ulceration. All animal experimental protocols and euthanasia criteria were approved by Stanford University’s Institutional Animal Care and Use Committee, known as the Administrative Panel on Laboratory Animal Care, which is accredited by the Association for Assessment and Accreditation of Laboratory Animal Care International.

### Tumor transplantation

B16F1 (#CRL-6323; ATCC) (referred to as B16 in this study), B16F10 (#CCL-6475; ATCC), B16-GM-CSF (Broz et al., 2014), B16-FLT3 (Broz et al., 2014), B16-OVA (Chen et al., 2023), and B16^Δ*Csf2*^ (generated in this study) cells were cultured in complete (supplemented with 10% fetal bovine serum, 1% penicillin–streptomycin, and 1% L-glutamine) DMEM (Corning). MC38 cells were cultured in complete RPMI medium (Corning). All cell lines were tested for *Mycoplasma* contamination periodically by PCR (Uphoff and Drexler, 2005). Tumors were implanted by inoculating 5 × 10^5^ cells subcutaneously in the right flank. For the BRaf/Pten model, melanoma lesions were induced by the topical application of 2 µl of 5 mM 4-hydroxytamoxifen (MilliporeSigma). Tumors were measured every other day, and the tumor volume was estimated using the formula [maximum dimension × (minimum dimension)^2^]/2.

### B16^Δ*Csf2*^ cell line generation

*Csf2* was knocked out in B16F1 cells (B16^Δ*Csf2*^) using CRISPR-Cas9. An single guide RNA (sgRNA) targeting the *Csf2* locus was selected from the Mouse Brie CRISPR knockout pooled library (Doench et al., 2016). DNA oligos (IDT) containing the following sgRNA sequence 5′-ATA​TTC​GAG​CAG​GGT​CTA​CG-3′ were annealed and ligated into lentiCRISPRv2. Lentivirus was produced by transfecting plasmid cocktails of (1) the transgene-expressing plasmid with ∆VPR (#8455; Addgene, gift from Bob Weinberg), (2) pCMV-VSV-G (#8454; Addgene, gift from Bob Weinberg) (Stewart et al., 2003), and (3) pAdVAntage (Promega) packaging plasmids into 293FT cells using TransIT-LT1 transfection reagent (cat#MIR 2300; Mirus). Transduced B16F1 cells were selected with puromycin at 5 μg/ml for 3 wk versus nontransduced controls to confirm selection. Cells were then single-cell-sorted into 96-well plates. Genotyping of resultant clones was performed by isolating genomic DNA from the cells and PCR to amplify the sgRNA-targeting site (see primers in Table S5), followed by Sanger sequencing the amplicons. The resulting amplicon sequences were compared with amplicon sequences obtained from nonedited B16F1 cells using the SYNTHEGO ICE analysis tool (https://ice.synthego.com). One clone encoding frameshift indels in the gene of interest was selected for use.

### Radiation therapy

Radiation was administered using an X-RAD SmART image–guided preclinical irradiator (Precision X-ray Inc.). Radiation treatment plans were optimized for each animal to ensure consistent dose distributions at a single dose of 20 Gy or three doses of 8 Gy each (3 × 8 Gy). B16, B16^Δ*Csf2*^, and MC38 tumors were treated when the volume reached 30–80 mm^3^, while the BRaf/Pten tumors were treated when their volume reached 100–200 mm^3^. For in vitro studies, B16 or MC38 cells were cultured as a monolayer and treated with 20 Gy of radiation using the Polaris SC-500 Series II orthovoltage irradiator (Kimtron) and cultured for 4 days before analysis for quantitative real-time PCR (qPCR) or ELISA.

### Cell depletion

T cell depletion was performed by injecting intraperitoneally 200 μg of Ab on days 1, 2, 5, 8, and 11 after RT, with either the following Abs produced in-house or obtained from BioXCell: αCD8 Ab (clone: 2.43), αCD4 Ab (clone: GK1.5), or isotype control IgG2b (clone: LTF-2). The *Foxp3*^DTR^ model was used for Treg depletion by inoculating intraperitoneally 50 and 25 ng DT (MilliporeSigma) per gram of body weight on days 3 and 6 after RT, respectively. Monocytes were depleted by inoculating intraperitoneally 20 μg of αCcr2 (clone: MC21) or IgG2b control Ab on days 1, 2, 3, 4, and 5 after RT. CD301b^+^ DCs were depleted using *Cd301b*^DTR^ mice inoculated with 50 ng DT intraperitoneally per gram of body weight on day 3 after RT, and 25 ng DT per gram of body weight on days 6, 9, and 11 after RT. For TAM depletion, mice were injected intraperitoneally with αCD115 (clone: AFS98) or IgG2a on day 1 (1 mg), and days 3 and 5 (0.5 mg) after RT. *Lyz2*^Cre+/+^ x*Csf1r*^LSL-DTR+/−^ (MM^DTR+^) or littermate control *Lyz2*^Cre+/+^ x*Csf1r*^LSL-DTR−/−^ (MM^DTR−^) mice were inoculated with 4 ng DT per gram of body weight intravenously on day 1 and intraperitoneally on days 3 and 5 after RT. DTR^−^ mice inoculated with DT, or DTR^+^ mice without DT inoculation were used as controls.

### Single-cell suspension

Tumors were harvested, and single-cell suspensions were obtained via enzymatic digestion using 400 U/ml of collagenase D (MilliporeSigma) and 50 μg/ml of DNase I (MilliporeSigma) in Hank’s balanced salt solution (HBSS) buffer with Ca^2+^ and Mg^2+^ (Corning) at 37°C for 60 min. 5 μM EDTA (Thermo Fisher Scientific) incubation for 5 min was used to stop the enzymatic digestion. LN cell suspensions were obtained by enzymatic digestion using 2.5 μg/ml Liberase TL (MilliporeSigma) and 50 μg/ml of DNase I in HBSS. Blood was obtained in 0.5 mM EDTA to prevent coagulation followed by lysis with ammonium–chloride–potassium lysis buffer (Gibco) to remove erythrocytes. All single-cell suspensions were filtered using a 70-μm strainer, and CD45^+^ cells were counted using CountBright Absolute Counting Beads (Thermo Fisher Scientific) and flow cytometry. For adoptive transfer, bone marrow monocytes were enriched from *Ccr2*^RFP^ × *Cx3cr1*^EGFP^ mice using the Miltenyi Biotec monocyte isolation kit (cat#130-100-629), and subsequently sorted as singlets, live, and RFP^high^ EGFP^+^. Sorted monocytes (5 × 10^5^ cells) were adoptively transferred via tail vein injection.

### Flow cytometry

Single-cell suspensions were incubated with Abs αCD16/32 (clone 2.4G2; produced in-house) at 4°C for 15 min followed by surface staining at 4°C for 20 min. For cytokine detection, tumor cell suspension was resuspended in R10 media (RPMI 1640 supplemented with 10% fetal bovine serum, 1% penicillin–streptomycin, 1% L-glutamine, and 0.1% 2-mercaptoethanol) and stimulated with phorbol 12-myristate 13-acetate (100 ng/ml), ionomycin (500 ng/ml), and brefeldin A (10 μg/ml) for 4 h followed by fixation and permeabilization in perm/wash buffer. For FOXP3 staining, cells were fixed with FOXP3 Transcription Factor fixation/permeabilization buffer. LSRFortessa X-20 (BD Biosciences) was used for acquisition and FlowJo for analysis. Abs used for flow cytometry are listed in Table S6.

### In vitro co-culture

CD4^+^ T cells were negatively enriched from LN of naïve B6 mice using an Ab cocktail generated in-house from hybridoma supernatants (αB220 [clone: RA36B2], αF4/80 [clone: HB198], αMHCII [clone: TIB120], αCD8 [clone: 2.43], and αNK1.1 [clone: PK136]) followed by sheep αRat Dynabeads, as described previously (Tadepalli et al., 2023). Following negative selection, naïve CD4^+^ T cells (CD4^+^ CD44^−^ CD45RB^+^) were sorted using a FACSAria Fusion (BD Biosciences) and stained with CellTrace Violet (CTV; Invitrogen). moDCs, TAMs, CD301b^+^, and CD301b^−^ myeloid DCs were sorted from B16 tumors on 5 days after RT, as shown in Fig. S3, A and B. 25,000 moDCs, TAMs, or maturing DCs were cultured with 75,000 naïve CD4^+^ T cells (1:3 ratio) in a 96-well U-bottom plate for 4 days. Cells were then harvested, stained, and analyzed using flow cytometry for CTV dilution.

### ELISA

Tumors were lysed using 50 mM Tris, pH 7.4, 250 mM NaCl, 5 mM EDTA, 50 mM sodium fluoride (NaF), 1 mM Na_3_VO_4_, 1% NP-40, 0.02% NaN_3_, and cOmplete EDTA-free protease inhibitor cocktail (1 tablet per 50 ml of solution; MilliporeSigma; list of reagents in Table S7). Tumor protein concentration was measured using Pierce BCA protein assay kit (Thermo Fisher Scientific) and normalized per 10 μg of total protein. GM-CSF was estimated using LEGEND MAX Mouse GM-CSF ELISA Kit, following the manufacturer’s instructions (BioLegend).

### CyTOF analysis

CyTOF analysis was performed as described previously (Tadepalli et al., 2023). Briefly, CD45-enriched single-cell tumor suspensions were stained with 0.25 μM cisplatin (Fluidigm) and CD45-barcoding Abs in CyFACS buffer (PBS with 2 mM EDTA and 1% bovine serum albumin). Samples were pooled, stained with primary Abs, and fixed using FOXP3/Transcription Factor staining buffer. Samples were stained with intracellular Abs and then incubated with 2% paraformaldehyde (Electron Microscopy Sciences) containing 125 nM indium intercalator overnight. Samples were then analyzed in a CyTOF2 (Fluidigm) at the Stanford Shared FACS Facility. For analysis, fcs files from CyTOF were imported into FlowJo. Mononuclear phagocytes were visualized via Uniform Manifold Approximation and Projection (UMAP) after removing lineage (T cells, B cells, natural killer cells, neutrophils, and eosinophils). Cell populations were identified using the FlowSOM algorithm.

### qPCR

RNA was extracted using TRIzol (Invitrogen) and quantified by NanoDrop. cDNA was synthesized using iScript Reverse Transcription Supermix (Bio-Rad), and qPCR was performed using iTaq Universal SYBR Green Supermix (Bio-Rad), following the manufacturer’s instructions. Primers (Table S5) were purchased from Integrated DNA Technologies. Data were analyzed by the 2^−ΔΔCT^ method, with mRNA levels plotted relative to the *Rpl13a* housekeeping gene.

### NanoString gene expression

Tumor-infiltrating DCs and TAMs were sorted from tumor single-cell suspensions. Sorted cells were resuspended at a concentration of 1,000–5,000 cells/μl in 1/3 RNeasy lysis buffer RLT, and analyzed using the Mouse Myeloid Innate Immunity V2 Standard Platforms (NanoString), following the manufacturer’s instructions. Samples were processed using NanoString Digital Analyzer followed by ROSALIND (https://rosalind.bio). DEGs were determined based on a log_2_(fold change) of 1.5 and an adjusted P value <0.05 for the specified comparisons. Pathway enrichment analysis was generated using ROSALIND, from the directed global significance scores.

### scRNAseq and analysis

CD45^+^ cells from tumor single-cell suspensions were enriched on days 0 and 5 after RT by positive selection using αCD45-Biotin and αBiotin magnetic microbeads (Miltenyi). Cells were sorted as CD45^+^CD19^−^CD3^−^NK1.1^−^Ly6G^−^ cells followed by the exclusion of eosinophils (CD24^+^ MHCII^−^) and pDCs (CD11b^−^CD24^−^), and processed using 10x Genomics. Cell Ranger software (10x Genomics) was employed for demultiplexing, barcode generation, and count matrix generation. Samples were analyzed using Seurat (version 3) in R4.3.1. Both treatment conditions were merged together for downstream analysis, filtering, and normalization. Cells were filtered based on criteria including the number of genes between 50 and 7,000, and mitochondrial genes <10%. The data were normalized using Seurat’s Normalize Data function (scale factor 10,000). Principal component analysis (PCA) was performed in Seurat, and the top 15 dimensions were analyzed using UMAP. Cell cycle scoring and regression were performed according to vignettes/cell_cycle_vignette.rmd, using the cell cycle profiles as previously described (Nestorowa et al., 2016). Specific myeloid populations of interest were selected and reclustered using Seurat’s Run UMAP function using the top 15 PCA dimensions.

### Analysis of human patient data

Human patient data were analyzed using the publicly available UCSC Xena platform (Goldman et al., 2020). The Cancer Genome Atlas data of patients with melanoma, glioblastoma, and head and neck cancer were analyzed. Human genes were selected based on the top 10 mouse genes from scRNAseq analysis of DC2s/moDCs or TAMs, which had a positive Pearson correlation coefficient within each population in human samples.

### Statistics

Prism software was used for statistical analysis. The average tumor growth curve was analyzed using a two-way ANOVA followed by Tukey’s multiple comparison post hoc test. The Kaplan–Meier survival curves were analyzed using the Mantel–Cox test. Comparisons between two groups were conducted using an unpaired *t* test, while comparisons between multiple groups were performed using one-way ANOVA with Tukey’s or Kruskal–Wallis’s post hoc tests. Nonsignificant results (P > 0.05) were not shown in the figures for clarity. *P ≤ 0.05, **P ≤ 0.01, and ***P ≤ 0.001.

### Online supplemental material

Fig. S1 shows T cell features after RT in B16 and BRaf/Pten tumors. Fig. S2 shows myeloid cell analysis after RT in B16 and BRaf/Pten tumors. Fig. S3 outlines cell purification strategies and gene signature assignment. Fig. S4 provides supporting data on the moDC function and transcriptome. Fig. S5 provides supporting information on the role of GM-CSF. Table S1 shows cellular signatures used in this study. Table S2 shows DEGs in each myeloid cluster, measured by scRNAseq. Table S3 shows DEGs in CD301b^+^ DC2s, on days 0 and 5 after RT. Table S4 shows normalized counts of DEGs between DC2s and TAMs on days 0 and 5 after RT, measured by NanoString. Table S5 shows list of primers used in this study. Table S6 shows list of Abs used in this study. Table S7 shows list of critical commercial reagents used in this study. Data S1 contains source data for all of the graphs presented in the figures.

## Supplementary Material

Table S1 shows cellular signatures used in this study.

Table S2 shows DEGs in each myeloid cluster, measured by scRNAseq.

Table S3 shows DEGs in CD301b^+^ DC2s on days 0 and 5 after RT.

Table S4 shows normalized counts of DEGs between DC2s and TAMs on days 0 and 5 after RT, measured by NanoString.

Table S5 shows list of primers used in this study.

Table S6 shows list of Abs used in this study.

Table S7 shows list of critical commercial reagents used in this study.

Data S1 contains source data for all of the graphs presented in the figures.

## Data Availability

NanoString data generated in this study are deposited in NCBI GEO (GSE243263). scRNAseq data generated in this study are deposited in NCBI GEO (GSE243502). CyTOF data generated in this study are deposited in FlowRepository (FR-FCM-Z6QN). No unique code was generated in this study. The B16^*ΔCsf2*^ cells are available upon request to J. Idoyaga.
